# Alterations in oral microbiome and salivary metabolome profiles associated with myocardial infarction: a case-control study

**DOI:** 10.1080/20002297.2026.2735104

**Published:** 2026-10-29

**Authors:** Shengnan Lei, Tuo Chen, Xiaodong Xie, Xu Zhang, Tianzhu Song, Ikram Khan, Zhiqiang Li

**Affiliations:** a School of Stomatology, Key Laboratory of Oral Disease, Northwest Minzu University, Lanzhou, Gansu, People's Republic of China; b Northwest Institute of Eco-Environment and Resources, Chinese Academy of Sciences, Lanzhou, Gansu, People's Republic of China; c University of Chinese Academy of Sciences, Beijing, People's Republic of China; d Department of Genetics, School of Basic Medical Sciences, Lanzhou University, Lanzhou, Gansu, People's Republic of China; e General Hospital of the People's Liberation Army Xizang Military, Lhasa, People's Republic of China; f School of Chemistry and Chemical Engineering, National Key Laboratory of Natural Product Chemistry, Lanzhou University, Lanzhou, Gansu, People's Republic of China

**Keywords:** Myocardial infarction, oral microbiota, salivary metabolomics, microbiome-metabolome interaction, biomarkers

## Abstract

**Background:**

Few studies have comprehensively profiled both the salivary microbiome and metabolome in myocardial infarction (MI). This case-control study therefore characterized microbial and metabolic alterations in MI patients and identified potential diagnostic signatures.

**Methods:**

Seventy participants (35 MI patients and 35 matched healthy controls) were enrolled. Salivary microbiota were profiled via 16S rRNA (V3–V4) sequencing, and metabolites were analyzed using untargeted LC-MS. Microbial diversity, taxonomic composition, and predicted functional pathways were assessed. Diagnostic performance was evaluated by ROC analysis, and microbiota–metabolite correlations.

**Results:**

Significant differences in oral microbial diversity were observed between MI patients and controls. MI patients exhibited significantly higher alpha diversity and a distinct community structure, with beta diversity separating between groups. Enriched taxa included *Bacteroidota*, *Veillonella*, and *Prevotella_7*, while *Streptococcus* and *Porphyromonas* were depleted in MI patients. A four-metabolite panel comprising elevated L-2-aminoadipic acid and reduced thromboxane B2, 4-oxo-2-nonenal, and arginyl-glutamate distinguished MI from controls with high diagnostic accuracy (AUC = 0.92–0.94). Pathway analysis implicated ferroptosis and arginine/proline metabolism as candidate mechanisms. Correlation analysis revealed a significant positive association between *Streptococcus* and L-2-aminoadipic acid.

**Conclusion:**

MI is associated with distinct oral microbial and salivary metabolomic signatures. A four-metabolite panel shows promise as a potential non-invasive biomarker signature, though external validation is required. Given the cross-sectional design, causality cannot be established.

## Background

Cardiovascular disease, particularly MI, remains a leading cause of global mortality, accounting for approximately 17.2 million deaths annually worldwide despite significant advances in prevention, early diagnosis, and interventional treatment [1,2]. Traditional risk factors, including hypertension, dyslipidemia, diabetes mellitus, smoking, and obesity, do not fully explain the observed burden of MI, suggesting that additional, non-traditional pathogenic drivers contribute to disease susceptibility and progression [3,4]. Among these emerging risk factors, chronic low-grade systemic inflammation has garnered considerable attention, as it plays a central role in all stages of atherosclerosis, from endothelial dysfunction and plaque formation to vulnerable plaque rupture and thrombotic occlusion [5,6]. This recognition has shifted research focus toward extra-cardiovascular sources of inflammation, particularly microbial ecosystems distant from the heart.

The oral cavity, the second most diverse microbial habitat in the human body after the gut, harbours over 700 bacterial species and represents a plausible and underappreciated contributor to cardiovascular pathology [7,8]. Epidemiological studies have consistently demonstrated a positive association between periodontitis, a chronic inflammatory disease triggered by oral dysbiosis, and increased risk of MI, with meta-analyses reporting odds ratios ranging from 1.3 to 2.5 after adjustment for traditional cardiovascular risk factors [9–12]. The causal link between periodontitis and cardiovascular disease has been formally recognised in consensus reports [13]. However, the mechanistic basis of this association remains incompletely understood. Proposed pathways include transient bacteremia, in which oral microbes or their virulence factors enter the circulation and directly colonise atherosclerotic plaques, and systemic diffusion of microbial metabolites or pathogen-associated molecular patterns (PAMPs), such as lipopolysaccharide (LPS), which may perpetuate low-grade endotoxemia and fuel inflammatory cascades [14–16].

Despite growing interest, prior research has been limited by a narrow focus on a handful of cultured pathogens, most notably *Porphyromonas gingivalis* and *Fusobacterium nucleatum*. Meanwhile, the vast majority of uncultivated or understudied oral taxa have been largely overlooked [7]. Recent multi-omics studies have begun to characterise the oral microbiome in MI patients. Hernández-Ruiz et al. compared the oral microbiome and inflammatory profiles in STEMI patients according to an inflammation-based risk score, revealing associations between specific taxa and systemic inflammatory markers [17]. Furthermore, Hernández-Ruiz and colleagues have also investigated the potential association of the oral microbiome with trimethylamine N-oxide (TMAO) quantification in Mexican patients with myocardial infarction, providing evidence for a link between oral microbial composition and this gut-derived metabolite implicated in cardiovascular risk [18]. Similarly, integrated microbiome and metabolome analyses have identified distinct oral microbial signatures in acute MI cohorts [9–12]. However, the functional potential of the oral microbiome, reflected in the metabolome, has received limited investigation.

Metabolites represent the downstream readout of microbial and host metabolic activities, and they can directly influence host physiology by modulating inflammation, oxidative stress, and platelet function [19–21]. Saliva, which is rich in both host-derived and microbe-derived metabolites, offers a uniquely non-invasive and readily accessible medium to capture these dynamic interactions in real time [22,23]. Saliva was selected as the sampling medium for this multi-omics study because it offers several advantages over other oral sampling methods: it is non-invasive, easily collected, requires minimal training, and reflects both host-derived and microbial metabolites from the entire oral cavity. In contrast, subgingival plaque provides site-specific information but requires specialised training for collection and may not capture the global oral microbial landscape [7]. However, saliva also presents notable limitations that warrant consideration. The presence of endogenous proteases, including salivary proteases such as kallikrein, cathepsin B, and matrix metalloproteinases, as well as bacterial-derived proteolytic enzymes, can significantly affect protein stability and degradation, potentially compromising proteomic analyses [24,25]. These proteolytic activities may alter the abundance of peptide and protein biomarkers, introduce variability in downstream analyses, and complicate the interpretation of protein-based findings. Additionally, salivary flow rate and composition can be influenced by circadian rhythms, hydration status, and diurnal variation, which may affect metabolite concentrations and microbial composition [26]. Furthermore, the dilution of systemic biomarkers in saliva can be substantial, necessitating sensitive analytical methods for accurate detection [27]. To mitigate these limitations, we implemented stringent pre-analytical protocols including immediate sample chilling, rapid centrifugation, and storage at −80 °C within 2 hours of collection, as detailed in the Methods section.

While integrated microbiome–metabolome analyses have revolutionised our understanding of the gut–brain and gut–liver axes, their application to the oral–cardiac axis has been remarkably limited [28–30]. Most studies have focused on blood or serum samples, with limited attention to saliva as a non-invasive medium for capturing both local and systemic metabolic signals. The gut–heart axis has been extensively studied through microbiome and metabolome integration [31]; the oral–heart axis remains comparatively underexplored, representing a significant knowledge gap. This proposed bidirectional communication highlights the potential of saliva as a non-invasive medium for exploring cardiovascular health.

To address this gap, we conducted a case–control study integrating 16S rRNA sequencing and untargeted LC-MS metabolomics to profile the oral microbiome and salivary metabolome of acute MI patients and matched healthy controls. We sought to define taxonomic shifts in the oral microbiome associated with MI, characterise salivary metabolomic alterations to identify potential diagnostic metabolites, map these metabolic changes to biological pathways, and explore correlations between dysbiotic taxa and discriminant metabolites. We hypothesised that MI is associated with a distinct oral microbial and metabolomic signature that could serve as a basis for the development of non-invasive diagnostic biomarkers. Saliva integrates host-derived and microbial metabolites, making it a non-invasive window into both compartments, though causal attribution requires further investigation.

## Materials and methods

### Study design and participant recruitment

This was a cross-sectional case-control study conducted at the General Hospital of the People's Liberation Army Xizang Military Area Command, Lhasa, China. Seventy participants, including 35 MI patients and 35 healthy controls, were recruited between March and October 2025.

*Inclusion criteria for MI patients*: (1) age ≥ 18 years; (2) diagnosis of ST-segment elevation myocardial infarction (STEMI) according to the Fourth Universal Definition of Myocardial Infarction [32]; (3) hospitalisation within 12 hours of symptom onset; (4) confirmed by elevated cardiac troponin I (>99th percentile) and electrocardiographic changes (ST-segment elevation ≥1 mm in ≥2 contiguous leads or new left bundle branch block); and (5) ability to provide informed consent.

Exclusion criteria for MI patients: (1) history of any chronic inflammatory or autoimmune disease (e.g. rheumatoid arthritis, inflammatory bowel disease, systemic lupus erythematosus); (2) active infectious diseases requiring systemic antibiotic therapy; (3) use of systemic antibiotics, including prophylactic or therapeutic courses, within the 3 months preceding hospitalisation; (4) use of systemic corticosteroids or other potent immunosuppressive or anti-inflammatory medications (e.g. methotrexate, cyclosporine, TNF-*α* inhibitors) within the 3 months preceding hospitalisation; (5) use of probiotic supplements within the 1 month preceding hospitalisation; (6) severe renal or hepatic impairment; (7) malignancy or ongoing oncological treatment; (8) pregnancy or lactation; and (9) inability to provide informed consent. Use of over-the-counter non-steroidal anti-inflammatory drugs (NSAIDs) for acute pain relief was not an exclusion criterion, but frequency and timing of use were recorded for potential confounding adjustment.

Healthy controls were recruited from the same geographical region through community health screenings and hospital staff volunteers. All controls underwent a comprehensive medical history review, physical examination, and 12-lead ECG to exclude undiagnosed cardiovascular disease. None had a history of MI, coronary artery disease, stroke, heart failure, or any chronic inflammatory condition. The same exclusion criteria regarding antibiotic, immunosuppressive, anti-inflammatory, and probiotic use within the preceding 3 months (or 1 month for probiotics) were applied to healthy controls.

A standardised questionnaire was administered to all participants to capture detailed information on medication use, including the type, dose, duration, and timing of any antibiotic, anti-inflammatory, immunosuppressive, or probiotic agents taken within the 6 months preceding sample collection. This information was used to assess potential confounding.

### Oral examination

The same oral examination protocol was applied to both MI patients and healthy controls. A single calibrated dentist performed comprehensive oral examinations immediately before saliva collection. Full-mouth periodontal examination was performed at six sites per tooth using a manual probe (UNC-15). Recorded parameters included bleeding on probing (BOP, %), expressed as the percentage of sites exhibiting bleeding within 15 seconds after probing. Periodontitis was classified according to the 2017 AAP/CDC case definition (mild/moderate/severe) based on clinical attachment loss, probing pocket depth, and radiographic assessment of alveolar bone loss. Radiographs were obtained to assess alveolar bone loss, defined as the distance from the cementoenamel junction to the alveolar bone crest.

For caries assessment, all permanent teeth (excluding third molars) were examined visually using the International Caries Detection and Assessment System II (ICDAS II) criteria https://www.icdas.org/. The ICDAS II system classifies caries lesions from Score 0 (sound surface) to Score 6 (extensive cavitation with exposed dentine). Caries was diagnosed at ICDAS II score ≥ 3 (enamel breakdown or more advanced lesions). The Decayed, Missing, and Filled Teeth (DMFT) index was calculated according to World Health Organisation criteria [33], with the D component including teeth with ICDAS II scores ≥ 3, M including teeth missing due to caries or periodontal disease, and F including teeth with permanent restorations without recurrent caries.

BOP was interpreted with caution in MI patients, as antiplatelet medications may influence gingival bleeding independent of periodontal inflammation [33]. No participant had received a dental cleaning in the preceding year or periodontal treatment within the past 3 months. The plaque index was initially recorded but was excluded from analysis because the time elapsed since the participants' last oral hygiene procedure was not recorded, limiting its reliability as a measure of habitual oral hygiene in the acute MI setting. Detailed oral hygiene habits (frequency of brushing, flossing, use of mouthwash) were not systematically assessed, representing a study limitation.

### Saliva sample collection

Saliva was collected on the first hospitalisation day before coronary interventions. Participants fasted for at least one hour. Unstimulated whole saliva was collected into separate sterile tubes for microbiome and metabolomic analyses. For microbiome analysis, 2 mL of saliva was collected into tubes prefilled with DNA preservation solution. For metabolomic analysis, 3 mL of saliva was collected into preservative-free tubes, immediately placed on ice, centrifuged at 4,000 × g for 10 minutes at 4 °C, aliquoted, and stored at −80 °C within 2 hours. Extraction blanks (phosphate-buffered saline) were included in each metabolomic batch; features detected in blanks were excluded. The same protocol was applied to controls.

### DNA extraction and bacterial 16S rRNA gene sequencing

Total genomic DNA was purified from 100 µL of each saliva sample following the standardised protocol for the TGuide S96 Magnetic Soil/Stool DNA Kit (Biomarker Technologies Co., Ltd., Beijing, China). Following extraction, DNA concentrations were determined using a high-sensitivity fluorometric assay (Qubit dsDNA HS Assay Kit, Invitrogen) to ensure accurate measurement. Extraction blanks and PCR negative controls were included in each batch; no amplicon was detected in negative controls. The final DNA eluates were stored at –80 °C to preserve stability for subsequent molecular analyses.

The hypervariable V3-V4 region of the bacterial 16S rRNA gene was amplified from genomic DNA using the universal primers 338F (5'-ACTCCTACGGGAGGCAGCA-3') and 806 R (5'-GGACTACHVGGGTWTCTAAT-3'). Primer pairs were modified with sample-specific Illumina index adaptors. PCR was performed in 10 µL reactions containing 5–50 ng DNA template, 0.3 µL of each primer (10 µM), 5 µL KOD FX Neo Buffer, 2 µL dNTPs (2 mM), and 0.2 µL KOD FX Neo polymerase. The thermocycling protocol consisted of initial denaturation at 95 °C for 5 min; 25 cycles of 95 °C for 30 s, 50 °C for 30 s, and 72 °C for 40 s; and a final extension at 72 °C for 7 min. Amplicons were purified with Agencourt AMPure XP beads, quantified fluorometrically (Qubit dsDNA HS Assay), pooled in equimolar ratios, and sequenced on an Illumina NovaSeq 6000 platform.

### Bioinformatic analysis

Bioinformatic processing was conducted on the BMK Cloud platform (Biomarker Technologies Co., Ltd.). Raw sequencing reads were processed to remove primers and low-quality bases using Cutadapt (v1.9.1) and Trimmomatic (v0.33), respectively. Paired-end reads were merged with FLASH (v1.2.11), and chimeric sequences were identified and removed with UCHIME (v8.1). High-quality sequences were obtained by filtering with USEARCH (v10.0) to discard sequences shorter than 100 bp or with an expected error rate >2%. Operational taxonomic units (OTUs) were clustered at 97% similarity and the Ribosomal Database Project (RDP) database (version 11.5). OTU clustering at 97% similarity was performed to maintain compatibility with previous studies in this field and with the RDP database, which was selected due to platform compatibility. While ASV-based approaches offer higher resolution, OTU-based analysis remains widely used and validated for comparative microbiome studies. Before diversity analysis, the OTU table was rarefied to the minimum sequencing depth (32,481 sequences per sample). Alpha diversity was measured using Chao1, Shannon, Simpson, and ACE indices. PERMANOVA was performed using the adonis2 function in R (vegan package) with Bray-Curtis dissimilarity as the distance metric to assess beta diversity. Relative abundance was assessed at the phylum and genus levels. LEfSe was selected as the primary method for differential abundance analysis as it is a standard and validated approach for microbiome biomarker discovery [34]. While additional methods such as ANCOM-BC could provide complementary insights, LEfSe remains widely used and accepted in oral microbiome studies. Microbial community function was predicted from the 16S data using PICRUSt2 and annotated against the KEGG database. Differences in functional pathways were assessed using Welch's t-test.

### Salivary metabolite extraction and LC-MS analysis

Frozen saliva supernatant aliquots (200 µL) were thawed on ice and mixed with 600 µL of ice-cold methanol:acetonitrile: water (2:2:1, v/v/v) containing isotopically labelled internal standards. The mixture was vortexed for 30 seconds, incubated at −20 °C for 1 hour to precipitate proteins, and centrifuged at 14,000 × g for 20 minutes at 4 °C. The supernatant was transferred to a clean tube and dried under a gentle stream of nitrogen gas at 30 °C. The dried extract was reconstituted in 100 µL of 50% aqueous acetonitrile containing 0.1% formic acid, vortexed for 30 seconds, and centrifuged at 14,000 × g for 10 minutes at 4 °C. The final supernatant (1 µL) was injected into the UHPLC system. Protein pellets were solubilized in 0.1 M NaOH, and protein concentration was measured using the BCA assay for normalisation purposes.

Untargeted metabolomic profiling was performed using a Waters Acquity I-Class PLUS UHPLC system coupled to a Xevo G2-XS QTof high-resolution mass spectrometer. Separation was achieved on an Acquity UPLC HSS T3 column (100 mm × 2.1 mm, 1.8 µm) maintained at 40 °C. The mobile phase consisted of (A) 0.1% formic acid in water and (B) 0.1% formic acid in acetonitrile. The gradient programme was as follows: 0–1 min, 1% B; 1–8 min, 1–99% B; 8–10 min, 99% B; 10–10.1 min, 99–1% B; and 10.1–12 min, 1% B. Total run time was 12 minutes. The injection volume was 1 µL. The mass spectrometer operated in MSe mode with electrospray ionisation (ESI) in both positive and negative polarities. Data were acquired in a single cycle with alternating low (2 V) and high (10–40 V) collision energies. Source parameters included a capillary voltage of ±2.0 kV (positive/negative), a cone voltage of 30 V, a source temperature of 150 °C, and a desolvation temperature of 500 °C. Gas flows were set to 50 L/h (cone) and 800 L/h (desolvation). Data acquisition was controlled by MassLynx software (v4.2).

The following isotopically labelled internal standards were added to each sample before extraction to monitor extraction efficiency and correct for matrix effects: L-phenylalanine-d5 (for amino acids), cholic acid-d4 (for bile acids), and 13C5-L-glutamic acid (for organic acids). All internal standards were purchased from Sigma-Aldrich. The coefficient of variation for internal standard peak areas was <15% across all samples, indicating consistent extraction performance.

### LC-MS data processing and metabolite annotation

Raw LC-MS data were processed using Progenesis QI software (v2.3, Waters Corporation). The processing workflow consisted of the following steps: (1) alignment: retention time alignment was performed using the QC sample as a reference to correct for retention time drift; (2) peak picking: features were detected with a minimum peak width of 5 seconds and a signal-to-noise ratio threshold of 5; (3) deconvolution and filtering: co-eluting peaks were deconvoluted using the software's isotope and adduct modelling algorithm; features with >50% missing values across samples were excluded; (4) blank subtraction: features detected in extraction blanks with a signal intensity >5% of that in the mean sample signal were removed to exclude background contaminants; and (5) data normalisation: feature intensities were normalised by total peak area, probabilistic quotient normalisation (PQN), and total protein concentration; results were consistent across methods and PQN-normalised data are reported in the main text. Missing values were imputed using the k-nearest neighbour algorithm (k = 5) in the impute R package.

Metabolite annotation was performed by matching accurate mass (mass error < 10 ppm) and retention time to (1) authentic standards (where available), (2) the Human Metabolome Database (HMDB, v4.0), (3) METLIN (v2.0), and (4) the in-house library of >500 standards. Confidence levels were assigned according to the Metabolomics Standards Initiative: Level 1 (confirmed by authentic standard), Level 2 (putatively annotated based on accurate mass and MS/MS fragmentation matching), Level 3 (putatively characterised compound class), and Level 4 (unknown). For differential metabolites reported in the main text, all were annotated at Level 2 or higher, with 4-oxo-2-nonenal confirmed by an authentic standard (Level 1).

### Statistical analysis

No formal power calculation was performed for this study. Sample size is comparable to published multi-omics studies [8,10–12,35–37]. Descriptive statistics were used to assess baseline characteristics; quantitative variables are presented as mean and standard deviation (±) for normally distributed variables and as median (IQR) for non-normally distributed variables, and categorical variables as percentages. Normality of continuous variables was assessed using the Shapiro–Wilk test. Group differences in clinical indices were assessed using the Student's t-test for normally distributed quantitative variables (age, BMI, systolic BP, diastolic BP, LDL, HDL, number of teeth, missing teeth, salivary flow rate, DMFT components, and BOP) or the nonparametric Mann–Whitney test for non-normally distributed variables (triglycerides). Categorical variables (sex, smoking, diabetes mellitus, periodontitis) were compared using the chi-square test. Spearman's correlation coefficient was used to assess relationships between oral microbiota and salivary metabolites and clinical parameters, visualised via the R package ‘pheatmap.’ For metabolomic data, we identified differential metabolites using OPLS-DA (VIP > 1), followed by univariate testing with Benjamini–Hochberg correction for multiple comparisons. Statistical significance was defined as a *p*-value of ≤ 0.05.

## Results

### Baseline characteristics of the participants

A total of 70 participants were enrolled, comprising 35 patients with MI and 35 healthy individuals. The demographic and clinical characteristics of the participants are summarised in Table 1. Among the clinical parameters, higher age, BMI, triglycerides, LDL-cholesterol, and lower HDL-cholesterol levels were observed in patients with MI than in healthy individuals. However, no significant differences were observed in demographic and clinical parameters between the groups.

**Table 1.** Demographic information, clinical parameters, and medical history of participants.

Characteristics	MI patients (*n* = 35)	Healthy individuals (*n* = 35)	*P*-value
Age (years)	43.65 ± 7.20	41.11 ± 6.85	0.135
Sex (Male/Female)	34/1	34/1	1.000
Body mass index (kg/m^2^)	27.4 ± 4.2	26.8 ± 4.0	0.542
Smoking (yes/no)	18/17	18/17	0.999
Diabetes mellitus (yes/no)	3/32	0/35	0.239
Systolic BP (mmHg)	128.97 ± 21.54	122.02 ± 15.61	0.126
Diastolic BP (mmHg)	90.51 ± 18.77	84.48 ± 13.70	0.116
Triglycerides (mmol/L)	2.48 ± 3.06	1.38 ± 0.72	0.165
LDL (mmol/L)	3.25 ± 1.29	3.05 ± 1.10	0.135
HDL (mmol/L)	0.86 ± 0.25	0.99 ± 0.20	0.113
**Medical management of MI patients**			
Aspirin loading (300 mg)	35/35 (100%)	-	-
Ticagrelor loading (180 mg)	35/35 (100%)	-	-
Statin (any)	35/35 (100%)	-	-
Beta-blocker	22/35 (63%)	-	-
ACEi/ARB	18/35 (51%)	-	-
Number of teeth	26.2 ± 3.5	27.1 ± 3.8	0.312
Missing teeth	3.8 ± 3.2	2.9 ± 3.0	0.241
Salivary flow rate (mL/min)	0.32 ± 0.11	0.35 ± 0.09	0.213
Periodontitis (yes/no)	14/21	11/24	0.452
**Periodontitis severity**			
Mild	6	5	-
Moderate	7	5	-
Severe	1	1	-
**DMFT index**			
Decayed (D)	1.8 ± 1.5	1.5 ± 1.3	0.391
Missing (M)	3.8 ± 3.2	2.9 ± 3.0	0.241
Filled (F)	0.9 ± 1.2	1.4 ± 1.6	0.152
DMFT total score	6.5 ± 4.2	5.8 ± 3.9	0.478
BOP (%)	28.5 ± 12.3	24.2 ± 11.5	0.139

Data are presented as mean and standard deviation. Student's t-test and the Mann–Whitney U test were applied for comparisons of clinical variables between two groups. Abbreviations: ACEi, angiotensin-converting enzyme inhibitor; ARB, angiotensin receptor blocker; BOP, bleeding on probing; DMFT, Decayed, Missing, Filled Teeth; SD, standard deviation.

### OTU distribution and microbial diversity

To characterise the oral microbial community structure in MI patients and healthy controls, we first assessed OTU distribution and sequencing depth. MI patients showed 12,923 unique OTUs versus 10,303 in controls, with 2,252 shared OTUs (Figure 1A). Rarefaction curves reached a plateau, indicating adequate sequencing depth (Figure 1B). These results confirm adequate sequencing depth and reveal a distinct, more diverse oral microbiota in MI patients.

**Figure 1.**
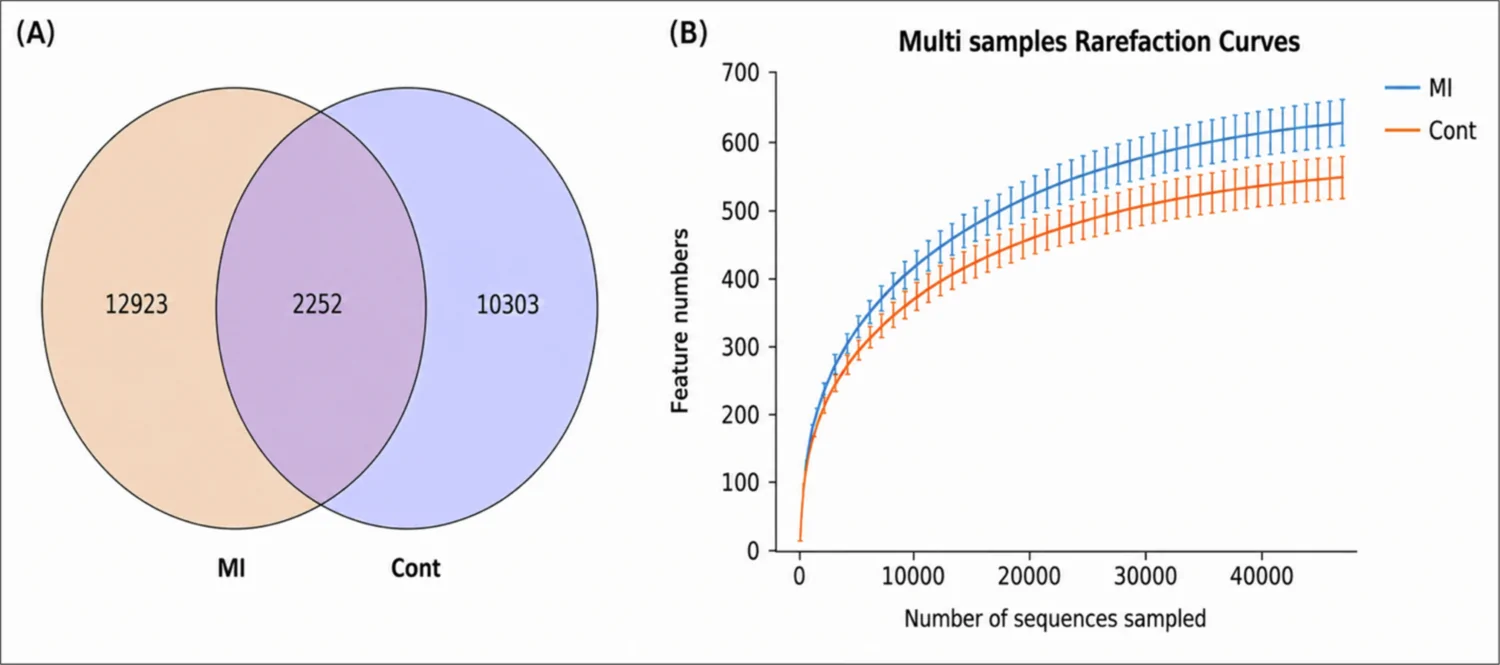
OTUs distribution and microbial sequencing depth between groups. (A) Venn diagram showing unique and shared OTUs between the MI and control groups. (B) Rarefaction curves showing sequencing depth.

To investigate the profile of the oral microbiota, we conducted 16S rRNA sequencing on the participants' saliva samples. The alpha diversity at the OTU level was determined with the Chao, ACE, Shannon, and Simpson indices. No significant differences were observed in the Chao1 and ACE indices between patients with MI and healthy individuals, indicating similar species richness between the two groups. However, the diversity indices, including Shannon and Simpson, were significantly higher in patients with MI than in healthy controls (Figure 2A–D). This finding suggests that MI is associated with greater microbial evenness and diversity within the oral cavity, reflecting a shift toward a more heterogeneous microbial community. The increased alpha diversity in MI patients may indicate a disruption of the normal oral microbial equilibrium, potentially driven by systemic inflammatory changes, medication effects, or altered oral environmental conditions associated with acute myocardial infarction. Notably, the Chao1 and ACE indices, which primarily measure species richness, did not differ significantly between groups, suggesting that the observed increase in Shannon and Simpson diversity is driven primarily by changes in species evenness rather than an increase in the total number of species present.

Beta diversity was measured with the Bray–Curtis distance, and the PCoA plot demonstrated distinct microbial community structures between groups (Figure 2E). Significant inter-group differences in microbial community structure were confirmed by PERMANOVA analysis of Bray-Curtis distances (*p* = 0.001; Figure 2F). However, the small effect size (R^2^ = 0.021) indicates that group-level differences explain only a modest fraction of community variation. This suggests that other unmeasured factors (e.g. diet, oral hygiene, host genetics) contribute substantially to inter-individual variation, and the clinical significance of these compositional differences should be interpreted with caution.

**Figure 2.**
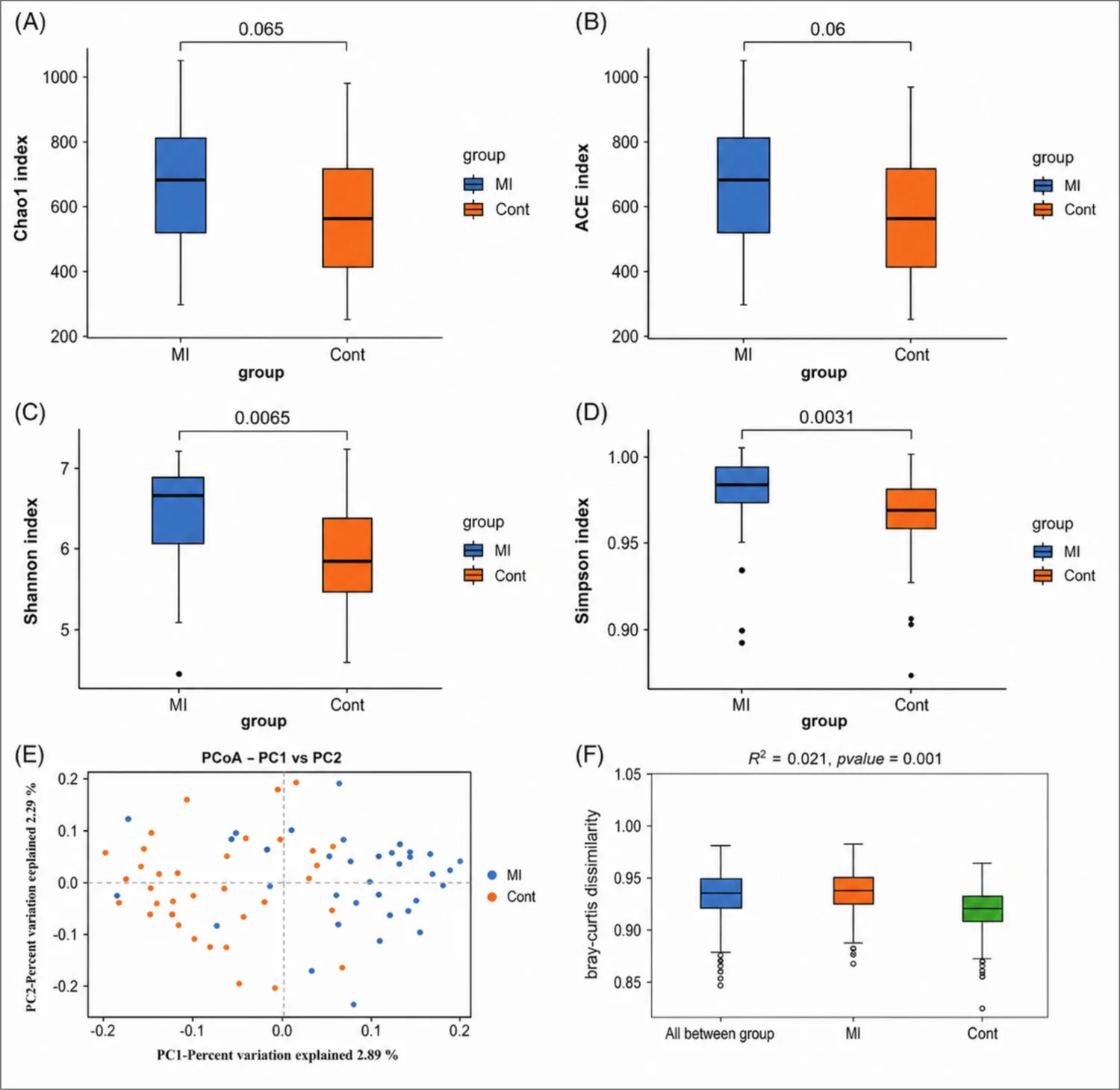
Oral microbiota diversity between patients with MI and Control groups. (A–D) Alpha diversity indices: (a) Chao index, (b) ACE index, (c) Shannon index, (d) Simpson index. Statistical significance was determined using the Mann–Whitney U test (*p* < 0.05). (E) PCoA result based on Bray-Curtis distance. (F) PERMANOVA test based on Bray–Curtis distance (*p* = 0.001, R^2^ = 0.021). 2.

### Taxonomic composition, microbiota markers, and predicted functional pathways

Distinct differences in oral microbiota composition were observed between patients with MI and healthy individuals. The oral bacterial community was composed predominantly of 10 bacterial phyla and genera. At the phylum level, MI patients showed higher relative abundance of *Bacteroidota*, *Fusobacteriota*, and *Actinobacteriota*, and lower abundance of *Firmicutes*, *Proteobacteria*, and *Patescibacteria* (Figure 3A). At the genus level, MI was enriched in *Veillonella, Prevotella_7, Neisseria, Prevotella, Alloprevotella, Fusobacterium*, and *Actinomyces*, while *Streptococcus, Porphyromonas*, and *Haemophilus* were depleted (Figure 3B).

**Figure 3.**
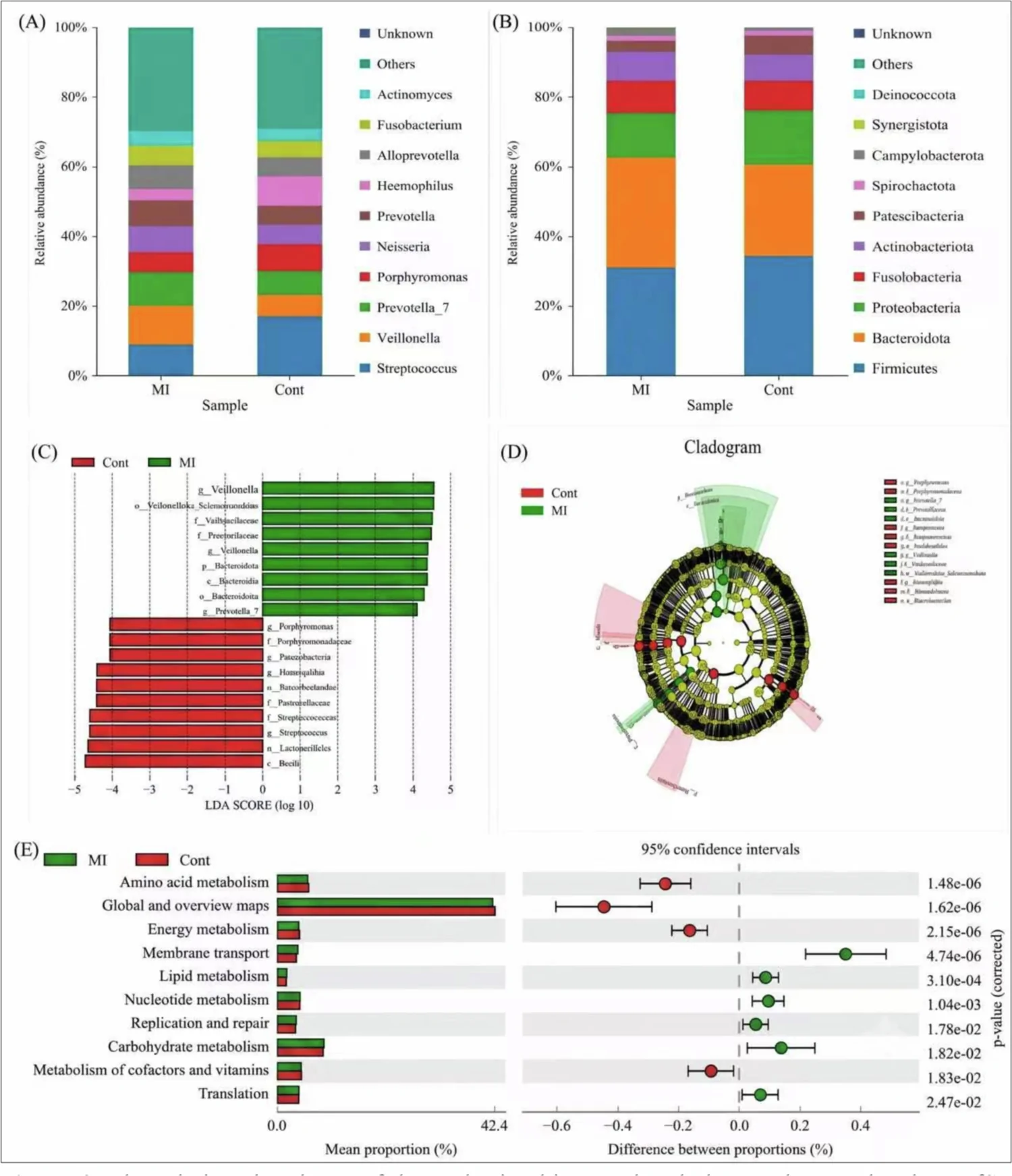
The relative abundance of the oral microbiota at the phylum and genus levels, LEfSe analysis, and predicted functional pathways. (A) The bar plot shows the relative abundance of bacterial phyla between MI patients and controls. (B) The relative abundance of bacterial genera. (C) LEfSe analysis shows significant differential taxa between both groups. (D) The cladogram illustrates the phylogenetic distribution of microbial taxa in each group. (E) Variations in functional pathways between patients with MI and healthy groups. Functional pathways were predicted from 16S rRNA sequencing data using PICRUSt2 and annotated against the KEGG database. Statistical significance was determined using Welch's t-test.

We further investigated potential oral bacterial biomarkers that distinguished patients with MI from healthy comparators. LEfSe analysis was used to identify distinct microbial signatures with significant differences in abundance across groups. The LDA scores indicated that nine species, including the phylum *Bacteroidota* and the genera *Veillonella* and *Prevotella_7, were enriched* in patients with MI, whereas 10 species were enriched in the healthy comparators (Figure 3C). The cladogram further illustrated the key phylogenetic lineages driving the compositional differences between patients with MI and healthy individuals (Figure 3D).

The functional profiles of the oral microbiota were annotated with the KEGG database.

Among the level 2 pathways with a relative abundance greater than 0.5%, the amino acid metabolism, energy metabolism, and metabolism of cofactors and vitamins were significantly upregulated in the MI group, whereas the membrane transport, lipid metabolism, replication and repair, carbohydrate metabolism, and translation metabolism pathways were markedly downregulated (Figure 3E).

### Salivary metabolomic profiling reveals distinct signatures in MI

To investigate the interplay between the oral microbiome and host metabolism, we performed untargeted metabolomic profiling on saliva samples from MI patients and controls. This analysis revealed substantial differences in the salivary metabolome. A volcano plot illustrated the distribution of 1,465 identified metabolites, with 738 upregulated and 727 downregulated in the MI group (Figure 4A). Among these, five metabolites, including 13Z, 16Z-Docosadienoic acid and L-2-Aminoadipic acid, were significantly upregulated, while SM (d19:1/PGJ2), Asparaginyl-Lysine, and Sorbaldehyde were considerably downregulated and showed correlation with clinical MI markers.

Principal Component Analysis (PCA) demonstrated a discernible separation between groups, with the first two principal components (PC1 = 30.29%, PC2 = 13.77%) accounting for a substantial portion of the metabolic variance (Figure 4B). The partial overlap observed reflects expected heterogeneity among MI patients, potentially attributable to clinical variables such as comorbidities or disease severity. To enhance group separation, a supervised Orthogonal Projections to Latent Structures-Discriminant Analysis (OPLS-DA) was employed, which clearly distinguished the MI metabolomic profile from that of controls (Figure 4C). We identified 10 metabolites that were significantly upregulated in the MI group compared to the controls (Figure 4D). Using stringent criteria, 20 distinct metabolites were identified. Key upregulated metabolites included ticagrelor, juvenile hormone III, and various complex lipids. Downregulated metabolites included seryltyrosine, the antioxidant gamma-L-glutamyl-L-cysteine, and pirfenidone (Figure 4E). Collectively, these results confirm a significant shift in the salivary metabolomic landscape following MI.

**Figure 4.**
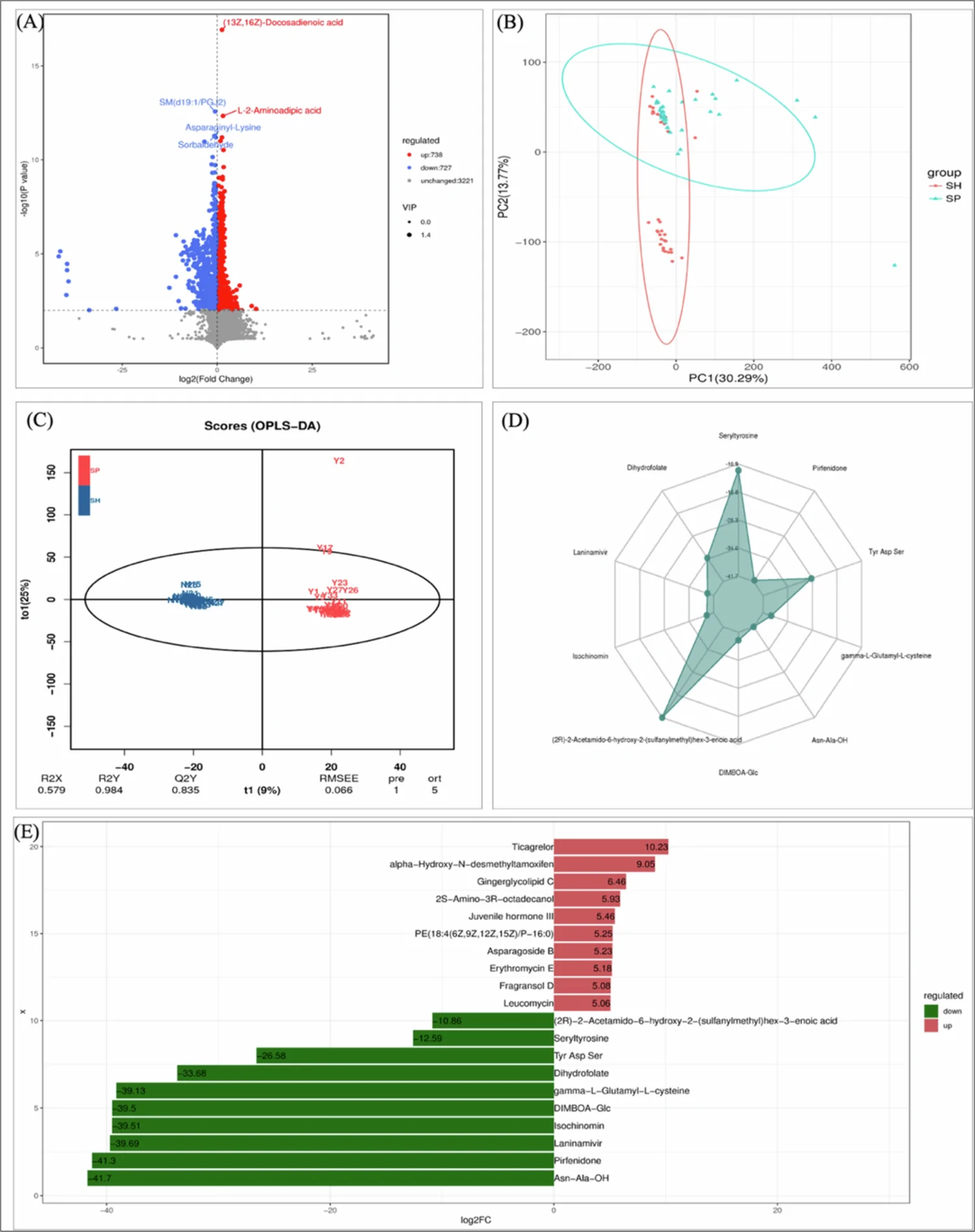
Salivary metabolomic profiling distinguishes MI patients from controls. (A) A volcano plot visualises differential metabolite abundance. (B) The PCA score plot demonstrates distance-based clustering and separation between MI and control groups. (C) The OPLS-DA score plot confirms separation between the MI and control groups. (D) The radar chart shows the top 10 upregulated metabolites in the MI group (E). The bar plot shows the top 20 significantly altered metabolite signatures in the MI group.

### Salivary metabolites as potential biomarkers for MI

To assess the diagnostic potential of the salivary metabolome, we performed receiver operating characteristic (ROC) analysis. Four metabolites demonstrated high discriminatory power, with area under the curve (AUC) values ranging from 0.92 to 0.94. Levels of L-2-Aminoadipic acid were significantly elevated in the MI group (*p* < 0.001), whereas 4-oxo-2-nonenal (*p* < 0.001), arginyl-glutamate (*p* = 0.002), and thromboxane B2 (*p* < 0.001) were decreased considerably (Figure 5). These results indicate a promising diagnostic signal in this discovery cohort, though AUC estimates may be optimistic without external validation.

**Figure 5.**
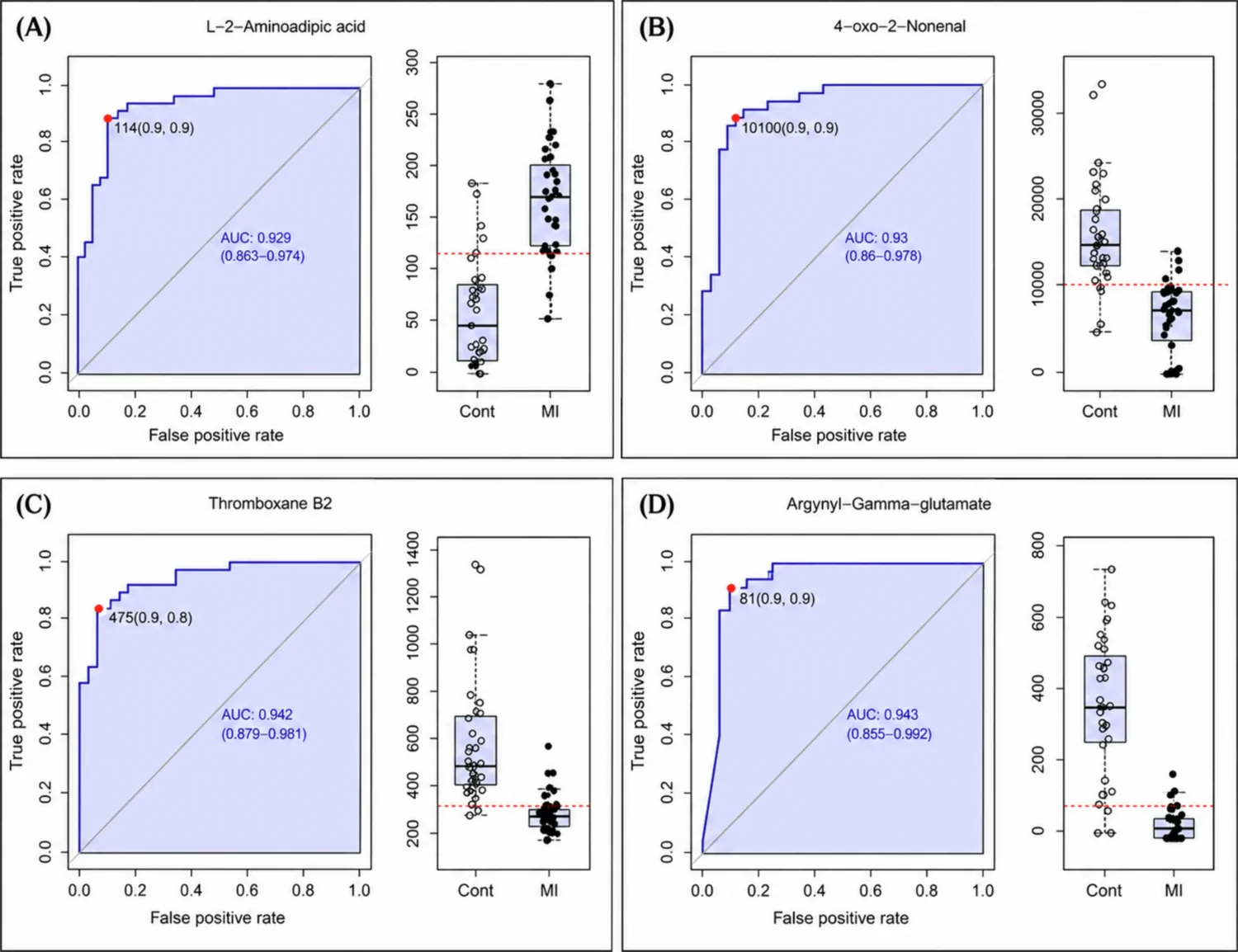
Diagnostic performance of salivary metabolites for MI. ROC analysis of differential metabolites that effectively discriminate MI patients from healthy controls.

### Functional variations in the salivary metabolome

To elucidate the functional implications of the altered salivary metabolome, we performed KEGG pathway enrichment analysis. Compared with healthy controls, MI patients exhibited significant perturbations in five metabolic and signalling pathways, including biotin metabolism, ferroptosis, arginine and proline metabolism, taste transduction, and FoxO signalling (Figure 6A).

**Figure 6.**
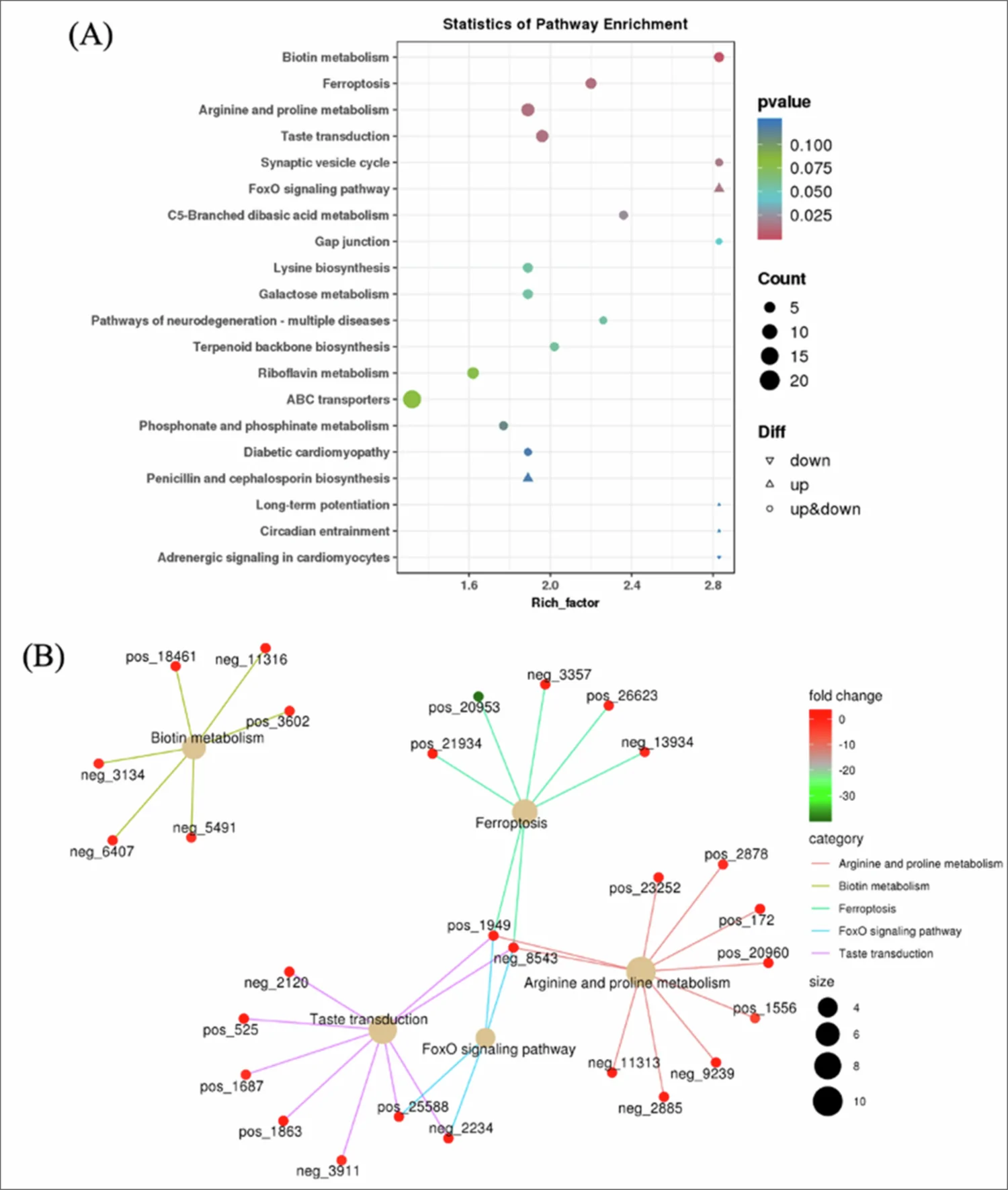
Altered salivary metabolome and predicted functional pathways in MI. (A) Bar plot of enriched KEGG pathways from metabolomic analysis shows significantly altered metabolic processes in MI patients compared to controls. (B) Network analysis illustrates the relationship between key metabolic pathways (light yellow nodes) and their associated differential metabolites. Node size corresponds to the number of metabolites per pathway, and colour intensity represents the average log2(fold change) of those metabolites.

To further explore the functional relationships among the differentially abundant metabolites, we performed KEGG pathway enrichment analysis and visualised the results using a network plot (Figure 6B). The analysis revealed that the altered metabolites in MI patients were significantly enriched in multiple metabolic pathways (Table 2). The network plot illustrated the interconnections among these pathways and their shared metabolites, highlighting several central hubs of metabolic perturbation.

**Table 2 .** Salivary metabolites linked to metabolic pathways relevant to MI pathophysiology.

Arginine and Proline metabolism	Taste transduction	Ferroptosis	Biotin metabolism	FoxO signalling pathway
Pos_23252: S-Adenosylmethioninamine	Pos_525: Aspartame	Pos_21934: Arachidonic acid	Pos_18461: 7,8-Diaminononanoate	Pos_25588: ADP
Pos_2878: L-Proline	Pos_1687: Acetylcholine	Pos_20953: gamma-L-Glutamyl-L-cysteine	Pos_3602: Biotin	Pos_1949: L-Glutamic acid
Pos_172: Spermine	Pos_1863: Sucrose	Pos_26623: 1-Octadecanoyl-2-(7Z,10Z,13Z,16Z-docosatetraenoyl)-sn-glycero-3-phosphoethanolamine	Neg_11316: Biocytin	Neg_8543: L-Glutamate
Pos_20960: N(omega)-Hydroxyarginine	Pos_25588: ADP	Pos_1949: L-Glutamic acid	Neg_3134: 8-Amino-7-oxononanoate	Neg_2234: D-Glucose
Pos_1556: gamma-Glutamyl-gamma-aminobutyraldehyde	Pos_1949: L-Glutamic acid	Neg_8543: L-Glutamate	Neg_5491: Dethiobiotin	
Pos_1949: L-Glutamic acid	Neg_8543: L-Glutamate	Neg_3357: (R)-Mevalonate	Neg_6407: 8-Amino-7-oxononanoic acid	
Neg_8543: L-Glutamate	Neg_2120: Maltose	Neg_13934: Adrenic acid		
Neg_2885: Trans-4-Hydroxy-L-proline	Neg_3911: Serotonin			
Neg_11313: S-Adenosyl-L-methionine	Neg_2234: D-Glucose			
Neg_9239: 4-Guanidinobutanamide				

### Correlation analysis of oral microbiota with clinical and metabolomic profiles

Associations between the top 5 most abundant species, clinical parameters, and metabolites were assessed through Spearman's correlation analysis. As an oral microbial biomarker for MI, no statistically significant associations were observed between oral microbial taxa and clinical parameters (Figure 7A). In contrast, *Streptococcus* showed a significant positive correlation with L-2-Aminoadipic acid metabolite (Figure 7B).

**Figure 7.**
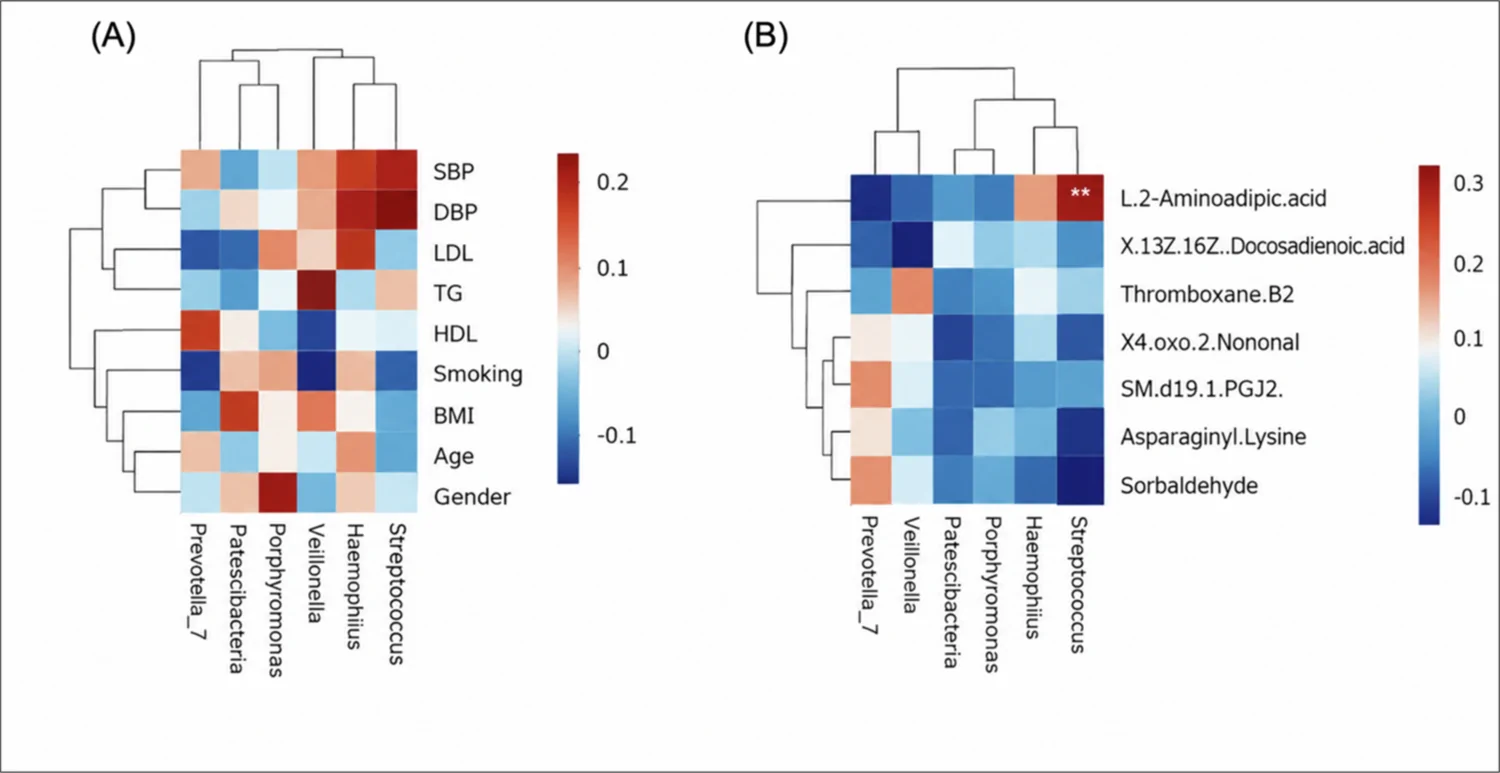
Associations between oral microbiota, clinical parameters, and salivary metabolites. (A) Correlation heatmap between oral bacterial taxa and clinical markers. (B) Correlation heatmap between selected oral bacterial taxa and significantly altered metabolites. Positive correlations are indicated in brown and negative correlations in blue. Statistical significance: *p < 0.05, **p < 0.01. Correlation coefficients and *p*-values were calculated using Spearman's correlation test.

## Discussion

This study integrated 16S rRNA sequencing and untargeted LC-MS metabolomics to characterise the oral microbiome and salivary metabolome of MI patients compared to healthy controls. We identified distinct microbial community shifts, altered alpha diversity, and a robust metabolomic signature that distinguished MI patients from controls with high diagnostic accuracy. Several previous studies have investigated oral microbial alterations in MI patients, reporting associations with genera such as *Veillonella, Streptococcus*, and *Prevotella* [8,10,12,38]; however, most focused on bacterial composition alone without integrating metabolomic data. Our study extends these findings by pairing microbial profiling with salivary metabolomics, revealing potential functional links between dysbiotic taxa and altered metabolites.

MI patients showed increased alpha diversity and distinct beta diversity. These findings align with a previous study [38], while some studies showed inverse results [8,10,12], discrepancies that may reflect heterogeneity in disease acuity, medication use, oral health status, and sequencing protocols. Nevertheless, altered diversity consistently supports oral dysbiosis in MI. Although the PERMANOVA analysis revealed a statistically significant difference in beta diversity between MI patients and controls (*p* = 0.001), the proportion of variance explained by group membership was modest (R^2^ = 0.021). This distinction is important, as statistical significance does not necessarily imply a strong separation between groups or a large effect size. The modest R^2^ value indicates that while MI status contributes to microbial community variation, other unmeasured factors likely play a more substantial role in shaping the oral microbiome [39,40].

The dysbiotic signature included enrichment of *Veillonella, Prevotella_7*, and Bacteroidota, and depletion of *Streptococcus* and *Porphyromonas*. *Veillonella* ferments lactate to propionate, potentially sustaining pro-inflammatory states, while *Prevotella* species induce Th17-mediated mucosal inflammation [41,42]. The Th17 pathway is a well-established driver of periodontal tissue destruction. Th17 cells produce interleukin-17 (IL-17), which promotes neutrophil recruitment and osteoclastogenesis, leading to alveolar bone resorption [43]. In periodontitis, Th17-driven mucosal inflammation is a hallmark of disease progression, and the presence of Th17-related biomarkers in periodontal tissues has been confirmed across multiple experimental studies [44]. Notably, Prevotella species have a marked capacity to drive Th17 immune responses through Toll-like receptor 2 activation, leading to production of Th17-polarising cytokines such as IL-23 and IL-1 from antigen-presenting cells [45]. This mechanism is biologically relevant to our findings, as the enrichment of Prevotella_7 in MI patients may contribute to a pro-inflammatory milieu that extends beyond the oral cavity. Beyond local ecology, these genera play important roles in the nitrate–nitrite–nitric oxide (NO) pathway, a major alternative source of NO bioavailability independent of the classical L-arginine–eNOS pathway [46]. Oral bacteria such as *Veillonella* and *Streptococcus* express nitrate reductases that reduce dietary nitrate to nitrite, which is subsequently reduced to NO in the acidic gastric environment and systemic circulation, contributing to vasodilation and blood pressure regulation [47]; *Prevotella* species have also been implicated in nitrate reduction [48]. The depletion of *Streptococcus* observed in our MI patients could therefore reduce NO bioavailability, contributing to endothelial dysfunction and hypertension, while enrichment of *Veillonella* might represent a compensatory response requiring functional metagenomic confirmation.

Although we performed a comprehensive periodontal examination, the periodontal parameters (PPD, CAL, BOP) and periodontitis severity did not differ significantly between MI patients and controls (Table 1). Correlation analysis revealed no significant associations between periodontal parameters and the relative abundance of differentially abundant taxa, nor between periodontitis severity and alpha or beta diversity indices. These findings suggest that the observed microbial differences between MI patients and controls are not primarily driven by periodontal disease severity, but rather reflect broader oral dysbiosis associated with MI status.

Interestingly, we found a lower abundance of *Porphyromonas*, contrasting with studies reporting enrichment of *P. gingivalis* in cardiovascular disease [49], a discrepancy that may reflect strain-level heterogeneity, community context, or medication effects, as statins have been shown to reduce anaerobic bacterial load [50]. Given its relevance as a key periodontal pathogen, the role of *P*. gingivalis in periodontitis and systemic disease warrants detailed consideration. *P*. gingivalis is a Gram-negative anaerobic bacterium that possesses multiple virulence factors, including gingipains (cysteine proteases), lipopolysaccharide (LPS), and fimbriae, which collectively contribute to periodontal tissue destruction [51]. Gingipains degrade host proteins, disrupt the complement system, and impair neutrophil function, facilitating bacterial survival and immune evasion [52]. The LPS of *P*. gingivalis is structurally distinct from that of other Gram-negative bacteria, acting as a weak agonist of TLR4 while effectively activating TLR2, which promotes a pro-inflammatory response through MyD88-dependent signalling [53]. Systemically, *P*. gingivalis has been implicated in atherosclerosis through multiple mechanisms, including induction of endothelial dysfunction, promotion of foam cell formation, and enhancement of platelet aggregation [54]. The presence of *P*. gingivalis DNA and antigens has been detected in human atherosclerotic plaques, suggesting direct bacterial involvement in vascular pathology [55]. The depletion of Porphyromonas observed in our cohort is noteworthy, as it contrasts with the well-established role of this pathogen in periodontal and cardiovascular diseases. This discrepancy may be attributed to the relatively mild periodontal disease status of our participants, as severe periodontitis is more consistently associated with *P*. gingivalis enrichment [56]. Alternatively, statin therapy, which was administered to all MI patients in our study, has been shown to inhibit the growth of *P*. gingivalis and reduce its virulence factors, potentially explaining the lower abundance observed in the MI group [57]. Notably, Tannerella forsythia, another key member of the red complex, has been linked to cardiovascular outcomes [58,59], though it was not among the significantly enriched or depleted taxa in our LEfSe analysis, likely due to limitations of 16S rRNA V3–V4 sequencing in resolving this fastidious anaerobe or the relatively mild periodontal disease in our participants. Periodontal severity did not show significant associations with microbial composition, likely due to limited sample size and inclusion of controls with periodontitis (31%), which attenuated group differences [57]. Moreover, statins may have contributed to the depletion of anaerobic taxa such as *Porphyromonas*, though we cannot isolate this effect from other therapies or the disease state itself. BOP interpretation in MI patients also requires caution, as antiplatelet and anticoagulant therapies may increase gingival bleeding propensity independent of the underlying inflammatory state.

The salivary metabolome showed elevated L-2-Aminoadipic acid (2-AAA), a metabolite linked to diabetes and coronary heart disease risk, and depleted gamma-L-Glutamyl-L-cysteine (*γ*-GC), a glutathione precursor [60,61], suggesting redox imbalance. A four-metabolite panel (2-AAA elevated; thromboxane B2, 4-oxo-2-nonenal, arginyl-glutamate depleted) demonstrated high discriminatory performance (AUC 0.92–0.94) [62,63]. The detection of ticagrelor in saliva confirms medication adherence in MI patients but may also independently influence the metabolomic signature. Thromboxane B2 depletion in saliva is notable, as it is typically elevated systemically in CVD [64,65], suggesting compartment-specific metabolic shifts. Though salivary thromboxane B2 may not directly mirror systemic levels, paired plasma–saliva studies are needed to resolve this. However, clinical translation of this panel requires external validation, medication-effect characterisation, and pre-analytical standardisation [27], and benchmarking against established tools such as troponin; the most plausible application is as a non-invasive screening tool in low-resource or pre-hospital settings, pending prospective evaluation.

Pathway analysis identified ferroptosis and arginine/proline metabolism as altered in saliva. The metabolites linked to ferroptosis (Table 2), arachidonic acid, adrenic acid, and *γ*-GC, are implicated in lipid peroxidation and glutathione metabolism, central processes in ferroptotic cell death, a known driver of ischaemia–reperfusion injury in cardiomyocytes [66–68]. In the arginine and proline metabolism pathway, L-proline, spermine, and S-adenosylmethionine were altered, suggesting disturbances in NO bioavailability and polyamine metabolism, with arginine metabolism disturbance contributing to endothelial dysfunction [69–71]. Salivary metabolites originate from both host systemic circulation and local oral microbial activity; thus, these discriminant metabolites likely reflect systemic metabolic shifts in MI that are detectable in saliva. While *P*. gingivalis was depleted in our cohort, this organism has been implicated in ferroptosis-related pathways through its effects on iron metabolism, oxidative stress, and lipid peroxidation; our study did not investigate this mechanism specifically, and its depletion, possibly related to statin use, precludes direct inference. Future studies combining metagenomic and functional analyses are needed to explore the interplay between periodontal pathogens and ferroptotic pathways in MI.

The pharmacological management of MI patients warrants careful consideration when interpreting our metabolomic findings. All patients received loading doses of aspirin and ticagrelor, and statin therapy was initiated upon admission. Statins modulate 2-AAA metabolism and reduce 4-oxo-2-nonenal through pleiotropic anti-inflammatory and antioxidant effects [66]; they inhibit the mevalonate pathway, potentially affecting 2-AAA production, and upregulate antioxidant defence systems, lowering 4-oxo-2-nonenal [67]. Thus, some metabolite differences may reflect acute medication effects rather than disease pathophysiology. Similarly, aspirin irreversibly inhibits cyclooxygenase-1, reducing thromboxane B2 [68], which may partially explain its depletion in saliva despite typical systemic elevation in CVD. This underscores the importance of considering medication history in metabolomic studies; future studies should include medication-naïve patients or carefully control for medication status to disentangle disease-related from treatment-related metabolic signatures.

Despite these significant findings, this study has several limitations. (i) The modest sample size (*n* = 35/group) limits statistical power, particularly for correlation analyses, and the metabolite panel requires validation in larger cohorts. (ii) Although we performed a comprehensive periodontal examination, the relatively mild periodontal disease burden in our cohort (mostly mild to moderate periodontitis, with only one severe case per group) may have limited our ability to detect associations between periodontal severity and microbial composition. The lack of significant correlation between periodontal parameters and microbial taxa suggests that the observed oral dysbiosis in MI patients is not simply a reflection of periodontal disease severity but may be influenced by systemic factors related to MI itself. (iii) 16S rRNA (V3–V4) sequencing lacks species-level resolution and provides only relative abundance data, as demonstrated by Castillo et al. [72]; targeted qPCR offers superior sensitivity for fastidious anaerobes, and future studies should employ qPCR, full-length 16S sequencing, or shotgun metagenomics for higher taxonomic and functional resolution. (iv) The cross-sectional and single-centre designs preclude causal inference and limit generalisability. (v) The only significant microbiota–metabolite correlation was a positive association between *Streptococcus* and 2-AAA; however, genus-level resolution precludes attribution to a specific species or mechanistic inference. (vi) Potential confounders such as diet, oral hygiene, and socioeconomic status were not fully accounted for. (vii) While LEfSe was used as the primary method for differential abundance analysis, complementary approaches such as DESeq2 should be applied in future studies with larger cohorts to further validate the microbial signatures identified in this study.

## Conclusion

In conclusion, the present study demonstrates that myocardial infarction is associated with distinct alterations in oral microbial composition and salivary metabolomic profiles. The four-metabolite panel (L-2-aminoadipic acid elevated; thromboxane B2, 4-oxo-2-nonenal, and arginyl-glutamate reduced) distinguished MI patients from controls with good accuracy, suggesting that saliva may serve as a potential non-invasive source of metabolite-based biomarkers. Altered pathways, including ferroptosis and arginine/proline metabolism, represent candidate mechanisms for further investigation, though causality remains to be established. These findings provide a foundation for future prospective studies of the oral–cardiac axis and underscore the need for external validation in larger, independent cohorts with standardised pre-analytical protocols.

## Data Availability

The 16S rRNA gene sequencing raw data have been submitted to NCBI and can be found at: http://www.ncbi.nlm.nih.gov/bioproject/1359138.

## References

[1] Shao C , Wang J , Tian J , et al. Coronary artery disease: from mechanism to clinical practice In: Wang M. , editor. Coronary Artery Disease: Therapeutics and Drug Discovery. Advances in Experimental Medicine and Biology (vol 1177). Singapore: Springer; 2020. pp. 1–36. doi: 10.1007/978-981-15-2517-9_1 32246442

[2] Khan I , Irfan M , Bacha AS , et al. Host–Microbiota metabolic interactions in atherosclerosis: oral, gut, and blood perspectives. Probiotics Antimicrob Proteins. 2026;1–8. doi: 10.1007/s12602-026-11054-2 42154390

[3] Joseph P , Leong D , McKee M , et al. Reducing the global burden of cardiovascular disease, part 1: the epidemiology and risk factors. Circ Res. 2017;121(6):677–694. doi: 10.1161/CIRCRESAHA.117.308903 28860318

[4] Zaheen M , Pender P , Dang QM , et al. Myocardial infarction in the young: aetiology, emerging risk factors, and the role of novel biomarkers. J Cardiovasc Dev Dis. 2025;12(4):148. doi: 10.3390/jcdd12040148 PMC12028068 40278206

[5] Gallucci G , Turazza FM , Inno A , et al. Atherosclerosis and the bidirectional relationship between cancer and cardiovascular disease: from bench to bedside-part 1. Int J Mol Sci. 2024;25(8):4232. doi: 10.3390/ijms25084232 PMC11049833 38673815

[6] Madaudo C , Coppola G , Parlati AL , et al. Discovering inflammation in atherosclerosis: insights from pathogenic pathways to clinical practice. Int J Mol Sci. 2024;25(11):6016. doi: 10.3390/ijms25116016 PMC11173271 38892201

[7] Willis JR , Gabaldón T . The human oral microbiome in health and disease: from sequences to ecosystems. Microorganisms. 2020;8(2):308. doi: 10.3390/microorganisms8020308 PMC7074908 32102216

[8] Khan I , Irfan M , Khan I , et al. Integrated oral microbiome and metabolome analysis unveils key biomarkers and functional pathway alterations in patients with acute myocardial infarction. Front Cell Infect Microbiol. 2025;15:1607845. doi: 10.3389/fcimb.2025.1607845 PMC12350251 40822584

[9] Maier T . Oral microbiome in health and disease: maintaining a healthy, balanced ecosystem and reversing dysbiosis. Microorganisms. 2023;11(6):1453. doi: 10.3390/microorganisms11061453 PMC10304820 37374955

[10] Lei S , Chen T , Zhou J , et al. Distinct blood and oral microbiome profiles reveal altered microbial composition and functional pathways in myocardial infarction patients. Front Cell Infect Microbiol. 2025;15:1506382. doi: 10.3389/fcimb.2025.1506382 PMC12034738 40297611

[11] Lei S , Khan I , Zhang X , et al. Assessing oral and toothbrush microbial profiles among high-altitude individuals with and without periodontal disease: a case-control study. BMC Oral Health. 2024;24(1):993. doi: 10.3389/fcimb.2025.1506382 PMC11344349 39182077

[12] Khan I , Khan I , Xie P , et al. Insights into the blood, gut, and oral microbiomes in Chinese patients with myocardial infarction: a case-control study. BMC Microbiol. 2025;25(1):226. doi: 10.1186/s12866-025-03878-9 PMC12008866 40253360

[13] Sanz M , Marco del Castillo A , Jepsen S , et al. Periodontitis and cardiovascular diseases: consensus report. J Clin Periodontol. 2020;47(3):268–288. doi: 10.1111/jcpe.13189 PMC7027895 32011025

[14] Rai V , Agrawal DK . The role of damage-and pathogen-associated molecular patterns in inflammation-mediated vulnerability of atherosclerotic plaques. Can J Physiol Pharmacol. 2017;95(10):1245–1253. doi: 10.1139/cjpp-2016-0664 28746820

[15] Zhu X , Huang H , Zhao L . PAMPs and DAMPs as the bridge between periodontitis and atherosclerosis: the potential therapeutic targets. Front Cell Dev Biol. 2022;10:856118. doi: 10.3389/fcell.2022.856118 PMC8915442 35281098

[16] Erridge C . The roles of pathogen-associated molecular patterns in atherosclerosis. Trends Cardiovasc Med. 2008;18(2):52–56. doi: 10.1016/j.tcm.2007.12.003 18308195

[17] Hernández-Ruiz P , Amezcua-Guerra LM , López-Vidal Y , et al. Comparative characterization of inflammatory profile and oral microbiome according to an inflammation-based risk score in ST-segment elevation myocardial infarction. Front Cell Infect Microbiol. 2023;13:1095380. doi: 10.3389/fcimb.2023.1095380 PMC9968971 36860987

[18] Hernández-Ruiz P , Escalona Montaño AR , Amezcua-Guerra LM , et al. Potential association of the oral microbiome with trimethylamine N‐Oxide quantification in Mexican patients with myocardial infarction. Mediators Inflamm. 2024;2024(1):3985731. doi: 10.1155/2024/3985731 PMC10898950 38415052

[19] Qian G , Ho JW . Challenges and emerging systems biology approaches to discover how the human gut microbiome impacts host physiology. Biophys Rev. 2020;12(4):851–863. doi: 10.1007/s12551-020-00724-2 PMC7429608 32638331

[20] Yang SY , Han SM , Lee JY , et al. Advancing gut microbiome research: the shift from metagenomics to multi-omics and future perspectives. J Microbiol Biotechnol. 2025;35:e2412001. doi: 10.4014/jmb.2412.12001 PMC12010094 40223273

[21] Ahmad P , Moussa DG , Siqueira WL . Metabolomics for dental caries diagnosis: past, present, and future. MSRv. 2025;44(3):454–490. doi: 10.1002/mas.21896 38940512

[22] Matsuoka M , Soria SA , Pires JR , et al. Natural and induced immune responses in the oral cavity and saliva. BMC Immunol. 2025;26(1):34. doi: 10.1186/s12865-025-00713-8 PMC12007159 40251519

[23] González Agurto M , Olivares N , Canedo-Marroquín G , et al. The intersection of the oral microbiome and salivary metabolites in head and neck cancer: from diagnosis to treatment. Cancers. 2024;16(20):3545. doi: 10.3390/cancers16203545 PMC11506592 39456639

[24] Hazare C , Bhagwat P , Singh S , et al. Diverse origins of fibrinolytic enzymes: a comprehensive review. Heliyon. 2024;10(5):2405–8440. doi: 10.1016/j.heliyon.2024.e26668 PMC10907686 38434287

[25] Ioannou P , Katsoulieris E , Afratis NA . Matrix dynamics and microbiome crosstalk: matrix metalloproteinases as key players in disease and therapy. Int J Mol Sci. 2025;26(8):3621. doi: 10.3390/ijms26083621 PMC12027064 40332093

[26] Groschl M , Rauh M , Dorr HG . Circadian rhythm of salivary cortisol, 17α-hydroxyprogesterone, and progesterone in healthy children. Clin Chem. 2003;49(10):1688–1691. doi: 10.1373/49.10.1688 14500602

[27] Hernandes VV , Barbas C , Dudzik D . A review of blood sample handling and pre‐processing for metabolomics studies. Electrophoresis. 2017;38(18):2232–2241. doi: 10.1002/elps.201700086 28543881

[28] Caradonna E , Abate F , Schiano E , et al. Trimethylamine-N-oxide (TMAO) as a rising-star metabolite: implications for human health. Metabolites. 2025;15(4):220. doi: 10.3390/metabo15040220 PMC12029716 40278349

[29] Heinrich-Sanchez Y , Vital M . Trimethylamine-N-oxide formation, the bacterial taxa involved, and intervention strategies to reduce its concentration in the human body. Ann Med. 2025;57(1):2525403. doi: 10.1080/07853890.2025.2525403 PMC12224733 40598778

[30] Wang Z , Kaplan RC , Burk RD , et al. The oral microbiota, microbial metabolites, and immuno-inflammatory mechanisms in cardiovascular disease. Int J Mol Sci. 2024;25(22):12337. doi: 10.3390/ijms252212337 PMC11594421 39596404

[31] Li C , Stražar M , Mohamed AM , et al. Gut microbiome and metabolome profiling in the framingham heart study reveals cholesterol-metabolizing bacteria. Cell. 2024;187(8):1834–1852. doi: 10.1016/j.cell.2024.03.014 PMC11071153 38569543

[32] McLaren J , de Alencar JN , Aslanger EK , et al. From ST-segment elevation MI to occlusion MI: the new paradigm shift in acute myocardial infarction. JACC: Advances. 2024;3(11):101314. doi: 10.1016/j.jacadv.2024.101314 PMC11492056 39435181

[33] Surma S , Lip GY . Periodontitis and atrial fibrillation. Eur J Prev Cardiol. 2025;zwaf626. doi: 10.1093/eurjpc/zwaf626 41061679

[34] Segata N , Izard J , Waldron L , et al. Metagenomic biomarker discovery and explanation. Genome Biol. 2011;12(6):R60. http://genomebiology.com/2011/11/6/R60. 10.1186/gb-2011-12-6-r60 PMC3218848 21702898

[35] Khan I , Khan I , Kakakhel MA , et al. Comparison of microbial populations in the blood of patients with myocardial infarction and healthy individuals. Front Microbiol. 2022;13:845038. doi: 10.3389/fmicb.2022.845038 PMC9176212 35694288

[36] Khan I , Panaiotov S , Attia KA , et al. Integrated analysis of blood microbiome and metabolites reveals key biomarkers and functional pathways in myocardial infarction. J Transl Med. 2025;23(1):797. doi: 10.1186/s12967-025-06165-3 PMC12269162 40671045

[37] Khan I , Khan I , Usman M , et al. Analysis of the blood bacterial composition of patients with acute coronary syndrome and chronic coronary syndrome. Front Cell Infect Microbiol. 2022;12:943808. doi: 10.3389/fcimb.2022.943808 PMC9577097 36268223

[38] Li YL , Chen BY , Feng ZH , et al. Roles of oral and gut microbiota in acute myocardial infarction. J Adv Res. 2025;74:319–332. doi: 10.1016/j.jare.2024.10.009 PMC12302727 39447641

[39] Gilbert JA , Blaser MJ , Caporaso JG , et al. Current understanding of the human microbiome. Nat Med. 2018;24(4):392–400. doi: 10.1038/nm.4517 PMC7043356 29634682

[40] Falony G , Joossens M , Vieira-Silva S , et al. Population-level analysis of gut microbiome variation. Sci. 2016;352(6285):560–564. doi: 10.1126/science.aad3503 27126039

[41] Yekani M , Dastgir M , Fattahi S , et al. Microbiological and molecular aspects of periodontitis pathogenesis: an infection-induced inflammatory condition. Front Cell Infect Microbiol. 2025;15:1533658. doi: 10.3389/fcimb.2025.1533658 PMC12095233 40406516

[42] Huang Y , Tang J , Cai Z , et al. Prevotella induces the production of Th17 cells in the colon of mice. J Immunol Res. 2020;2020(1):9607328. doi: 10.1155/2020/9607328 PMC7657696 33204736

[43] Abusleme L , Moutsopoulos NM . IL‐17: overview and role in oral immunity and microbiome. Oral Dis. 2017;23(7):854–865. doi: 10.1111/odi.12598 PMC5398954 27763707

[44] Sidharthan S , Gopalakrishnan D , Kheur S , et al. Assessment of the role of Th17 cells and related biomarkers in periodontitis: a systematic review. Arch Oral Biol. 2025;175:106272. doi: 10.1016/j.archoralbio.2025.106272 40359716

[45] Larsen JM . The immune response to prevotella bacteria in chronic inflammatory disease. Immunology. 2017;151(4):363–374. doi: 10.1111/imm.12760 PMC5506432 28542929

[46] Lundberg JO , Gladwin MT , Weitzberg E . Strategies to increase nitric oxide signalling in cardiovascular disease. Nat Rev Drug Discovery. 2015;14(9):623–641. doi: 10.1038/nrd4623 26265312

[47] Silva-Cunha M , Lacchini R , Tanus-Santos JE . Facilitating nitrite-derived S-Nitrosothiol formation in the upper gastrointestinal tract in the therapy of cardiovascular diseases. Antioxidants. 2024;13(6):691. doi: 10.3390/antiox13060691 PMC11200996 38929130

[48] Yang Z , Du C , Chang Z , et al. Role of oral and gut microbiomes in enterosalivary nitrate metabolism and their effects on systemic disease. Front Cell Infect Microbiol. 2025;15:1612223. doi: 10.3389/fcimb.2025.1612223 PMC12267184 40677520

[49] Curia MC , Pignatelli P , D’Antonio DL , et al. Oral porphyromonas gingivalis and fusobacterium nucleatum abundance in subjects in primary and secondary cardiovascular prevention, with or without heterozygous familial hypercholesterolemia. Biomedicines. 2022;10(9):2144. doi: 10.3390/biomedicines10092144 PMC9496065 36140246

[50] Petit C , Batool F , Bugueno IM , et al. Contribution of statins towards periodontal treatment: a review. Mediators Inflamm. 2019;2019(1):6367402. doi: 10.1155/2019/6367402 PMC6415285 30936777

[51] Guo Y , Wang Y , Wang Y , et al. Heme competition triggers an increase in the pathogenic potential of porphyromonas gingivalis in porphyromonas gingivalis-candida albicans mixed biofilm. Front Microbiol. 2020;11:596459. doi: 10.3389/fmicb.2020.596459 PMC7738433 33343538

[52] Hajishengallis G , Diaz PI . Porphyromonas gingivalis: immune subversion activities and role in periodontal dysbiosis. Curr Oral Health Rep. 2020;7(1):12–21. doi: 10.1007/s40496-020-00249-3 PMC7747940 33344104

[53] Haritha A , Jayakumar A . Syndromes as they relate to periodontal disease. Periodontol 2000. 2011;56(1):65. doi: 10.1111/j.1600-0757.2010.00363.x 21501237

[54] Schenkein HA , Papapanou PN , Genco R , et al. Mechanisms underlying the association between periodontitis and atherosclerotic disease. Periodontol 2000. 2020;83(1):90–106. doi: 10.1111/prd.12304 32385879

[55] Gaetti-Jardim JE , Marcelino SL , Feitosa AC , et al. Quantitative detection of periodontopathic bacteria in atherosclerotic plaques from coronary arteries. J Med Microbiol. 2009;58(12):1568–1575. doi: 10.1099/jmm.0.013383-0 19679682

[56] de Coo A , Cruz R , Quintela I , et al. Genome‐wide association study of stage III/IV grade C periodontitis (former aggressive periodontitis) in a spanish population. J Clin Periodontol. 2021;48(7):896–906. doi: 10.1111/jcpe.13460 33745150

[57] Kamińska M , Aliko A , Hellvard A , et al. Effects of statins on multispecies oral biofilm identify simvastatin as a drug candidate targeting porphyromonas gingivalis. J Periodontol. 2019;90(6):637–646. doi: 10.1002/JPER.18-0179 PMC6545270 30506795

[58] Moscoso SB , Mendoza F , Castillo DM , et al. Periodontitis progression during cardiac rehabilitation after acute coronary syndrome: cohort study. Oral Dis. 2025;31(11):3167–3178. doi: 10.1111/odi.15382 40364535

[59] Håheim LL , Schwarze PE , Thelle DS , et al. Low levels of antibodies for the oral bacterium tannerella forsythia predict cardiovascular disease mortality in men with myocardial infarction: a prospective cohort study. Med Hypotheses. 2020;138:109575. doi: 10.1016/j.mehy.2020.109575 32088522

[60] Lee HJ , Jang HB , Kim WH , et al. 2-Aminoadipic acid (2-AAA) as a potential biomarker for insulin resistance in childhood obesity. Sci Rep. 2019;9(1):13610. doi: 10.1038/s41598-019-49578-z PMC6754510 31541119

[61] Hubert M. Investigating the Effects of 2-Aminoadipic Acid on Beta-Cell and Human Islet Function. 2025. doi: 10.7939/r3-x1e6-qx71

[62] Church L , Franks K , Medara N , et al. Impact of oral hygiene practices in reducing cardiometabolic risk, incidence, and mortality: a systematic review. Int J Environ Res Public Health. 2024;21(10):1319. doi: 10.3390/ijerph21101319 PMC11508105 39457293

[63] Dame ZT , Aziat F , Mandal R , et al. The human saliva metabolome. Metabolomics. 2015;11(6):1864–1883. doi: 10.1007/s11306-015-0840-5

[64] Rade JJ , Kronsberg SS , Kickler TS , et al. Association of systemic thromboxane generation with risk of developing heart failure. J Am Coll Cardiol. 2025;85(1):58–70. doi: 10.1016/j.jacc.2024.09.010 PMC12231177 39779056

[65] Liu Y , Liu Y , Liang F , et al. Association of thromboxane generation with the bleeding events in aspirin users. Platelets. 2025;36(1):2473953. doi: 10.1080/09537104.2025.2473953 40113268

[66] Piatek K , Feuerstein A , Zach V , et al. Nitric oxide metabolites: associations with cardiovascular biomarkers and clinical parameters in patients with HFpEF. ESC heart failure. 2022;9(6):3961–3972. doi: 10.1002/ehf2.14116 PMC9773705 35979962

[67] Mommersteeg PM , Schoemaker RG , Eisel UL , et al. Nitric oxide dysregulation in patients with heart failure: the association of depressive symptoms with L-arginine, asymmetric dimethylarginine, symmetric dimethylarginine, and isoprostane. Biopsychosoc Sci Med. 2015;77(3):292–302. doi: 10.1097/PSY.0000000000000162 25790241

[68] Monnier VM , Nemet I , Sell DR , et al. Transition metals and other forms of oxidative protein damage in renal disease. In: Studies on Renal Disorders 2010 Nov 22. Totowa, NJ: Humana Press. p. 25–50. doi: 10.1007/978-1-60761-857-7_2

[69] Zheng J , Conrad M . Ferroptosis: when metabolism meets cell death. Physiol Rev. 2025;105(2):651–706. doi: 10.1152/physrev.00031.2024 39661331

[70] Huang W . Ferroptotic Pathways and Iron Dynamics in Gastrointestinal Inflammation and Wound Healing (Doctoral dissertation). 2025. doi: 10.7302/25682

[71] Wang H , Xie Y . Advances in ferroptosis research: a comprehensive review of mechanism exploration, drug development, and disease treatment. Pharmaceuticals. 2025;18(3):334. doi: 10.3390/ph18030334 PMC11944624 40143112

[72] Castillo DJ , Rifkin RF , Cowan DA , et al. The healthy human blood microbiome: fact or fiction? Front Cell Infect Microbiol. 2019;9:148. doi: 10.3389/fcimb.2019.00148 PMC6519389 31139578

